# Efficient boosting of Omicron-reactive memory B cells after breakthrough infection protects from repeated exposure

**DOI:** 10.1016/j.isci.2025.112278

**Published:** 2025-03-25

**Authors:** Qingfei Chu, Kang Li, Qianxin He, Li Ren, Jiguo Wang, Shuo Wang, Xiaojing Liu, Ying Liu, Jiangshan He, Dan Li, Yiming Shao

**Affiliations:** 1 State Key Laboratory for Diagnosis and Treatment of Infectious Diseases, National Clinical Research Center for Infectious Diseases, National Medical Center for Infectious Diseases, Collaborative Innovation Center for Diagnosis and Treatment of Infectious Diseases, The First Affiliated Hospital, Zhejiang University School of Medicine, Hangzhou 310003, China; 2 National Key Laboratory of Intelligent Tracking and Forecasting for Infectious Diseases, National Center for AIDS/STD Control and Prevention, Chinese Center for Disease Control and Prevention, Beijing 102206, China; 3 Guangxi Key Laboratory of AIDS Prevention and Treatment, Guangxi Medical University, Nanning 530021, China; 4 Toroivd Technology Company Limited, Shanghai 200439, China; 5 College of Life Sciences, Beijing Normal University, 19 Xinjiekouwai Avenue, Beijing 100875, China; 6 Changping Laboratory, Beijing 102299, China

**Keywords:** Immunology, Cell biology

## Abstract

Exploring the impact of persistent mutations in SARS-CoV-2 variants and reduced immunity on breakthrough infections (BTIs) is crucial, particularly in understanding how antigen-specific memory B cells (MBCs) respond to new variants. We followed 107 participants who received the ancestral inactivated vaccine and experienced one or two Omicron BTIs over six months. Using flow cytometry, SARS-CoV-2 antigen probes, single-cell RNA sequencing, and B cell receptor (BCR) profiling, we assessed MBCs and immune diversity. Our findings revealed that although neutralizing antibody levels decreased over time, the number of specific MBCs remained stable and matured progressively. Notably, pre-existing Omicron-specific MBCs played a key role in preventing secondary Omicron infections. Differential gene analysis showed enrichment in antigen processing and immune regulation pathways, while clonal lineage analysis revealed more B cell expansion and V(D)J gene-specific rearrangements in high neutralization samples. These results emphasize MBCs’ critical role in long-term immunity and inform future vaccination strategies.

## Highlights

• Moderate group have higher and broader SARS-CoV-2-specific MBCs responses
• Moderate group exhibits stronger cross-reactive MBCs responses
• SARS-CoV-2-specific MBCs play a potential role in preventing secondary BTI
• B cell expansion and V(D)J rearrangements in the high neutralization group

## Graphical abstract

**Figure:**
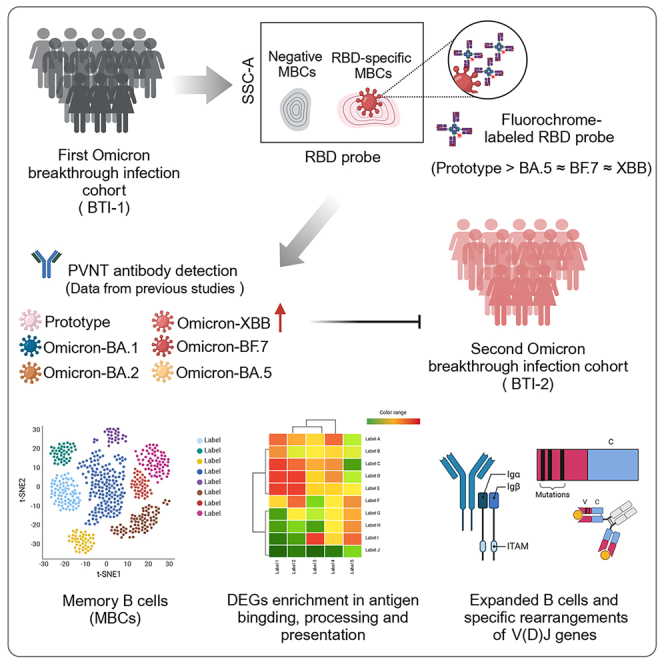

## Introduction

Since the beginning of the COVID-19 pandemic in 2019, SARS-CoV-2 has shown an incredible plasticity and led to waves of breakthrough infection (BTI) since late 2022 in China.^1^^,^^2^^,^^3^ Human immune system continuously adapts to the virus upon repeated exposure and respond to the newly emerging variants through pre-existing cross-reactive immunity. The elicit of Omicron-neutralizing antibodies (nAbs) are considered correlates of protection against recent infection and reflects an expansion of MBCs.^4^^,^^5^

Memory B cells (MBCs) are able to produce a secondary immune response upon exposure to antigens.^6^^,^^7^ In the context of a secondary infection, MBCs could proliferate and differentiate into antibody-secreting cells, thus dominating humoral recall responses initiated by similar antigen and mediating immediate and effective protection to the host.^8^ BTI induces B cell responses, increasing the size and breadth of antigen recognition.^9^^,^^10^^,^^11^ Previous studies have revealed that the higher frequency of MBCs produced after vaccination can predict the protection against sporadic SARS-CoV-2 infection.^12^ However, studies of long-lasting specific memory B and cross-reactive B cells in fully vaccinated individuals after Omicron BTI remain underdeveloped. There is also limited understanding of the in-depth impact of humoral immune responses after multiple Omicron BTI. Moreover, to what extent the first Omicron BTI-generated MBCs protects from future antigenic matched or mismatched variants remains an open question.

To address these questions, we characterize the impact of Omicron BTI on the intensity and breadth of specific MBCs and conduct correlation analysis with the nAbs against multiple strains obtained in the early stage. We also conducted specific B cell testing on people who had BTI six months after vaccination, mainly for the cohort that experienced BTI from December 2022 to January 2023, during which time the BA.5 and BF.7 lineages dominated in China. In addition, we selected high and low neutralization samples for single-cell RNA sequencing and B cell receptor (BCR) immune repertoire studies to further explore the evolution of the immune characteristics of the population after BTI. Since understanding the antigen-specific B cell memory pool is a key determinant of an individual’s ability to respond to emerging variants, our data will help guide the development of the next generation of vaccines.

## Results

### Study design and characteristics of cohort

**Figure 1:**
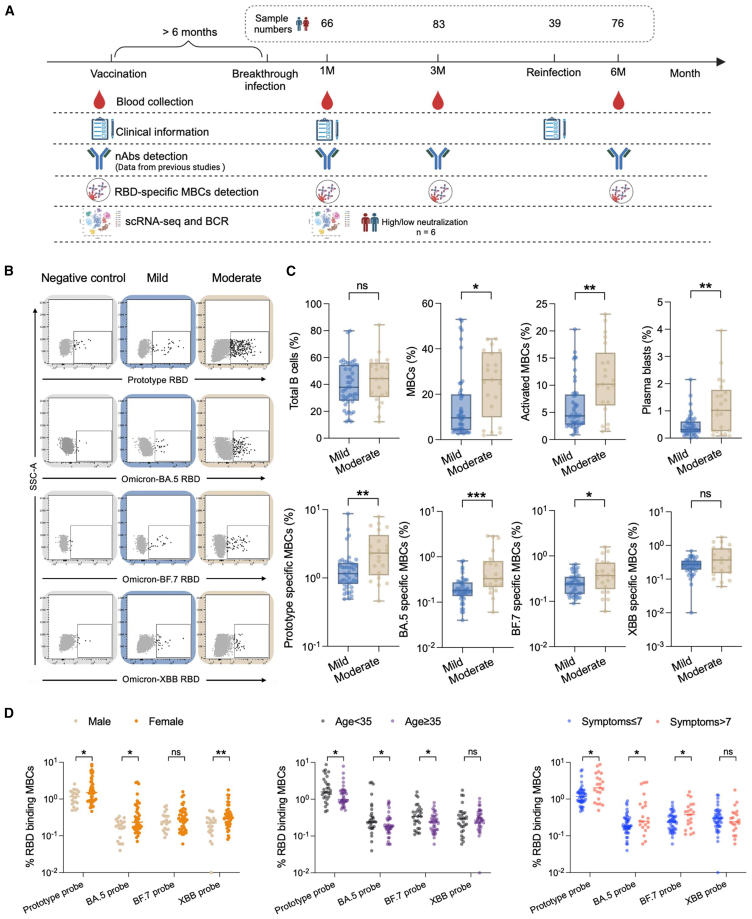
Moderate group have higher and broader SARS-CoV-2-specific MBCs responses (A) Flowchart of this study. (B) Schematic diagram of four RBD-specific MBCs (prototype RBD, Omicron-BA.5 RBD, BF.7 RBD, and XBB RBD) in the mild and moderate groups. Samples of healthy donors before the outbreak of COVID-19 were taken as negative control. They have neither been vaccinated with the COVID-19 vaccine nor been infected with SARS-CoV-2. (C) Comparison of total B, MBCs, activated MBCs (CD38^+^), plasma blasts (CD38^hi^), and four probe-specific MBCs in the mild and moderate groups at 1 month after BTI. Data are represented as median and interquartile range (IQR). (D) Comparison of the four probe-specific MBCs of different genders, ages and symptoms at 1 month after BTI. Data are represented as median. Comparisons between two groups were performed using the two-tailed non-parametric Mann-Whitney test. ^∗^*p* < 0.05; ^∗∗^*p* < 0.01; ^∗∗∗^*p* < 0.001; ns, no significance.

To understand the impact of Omicron BTI on MBCs and whether specific responses against its neoepitopes occur, we analyzed SARS-CoV-2-specific MBCs responses in 107 subjects both cross-sectionally (different clinical symptoms) and longitudinally (different time points). These individuals were sampled at three time points (1, 3, and 6 months) after BTI to fully characterize B cell responses from early extrafollicular responses to later long-term memory precipitation, combining early neutralizing antibody data with flow cytometry results and single cell sequencing transcriptome and BCR immune repertoire data (Figure 1A).

Based on prevailing variants during December 2022 to January 2023 in China (data obtained from https://gisaid.org/phylodynamics/china-cn/), these 107 participants were all experienced Omicron-BA.5 and/or BF.7 BTI. With an average age of 37 (range: 8–66) years, comprising 39% males. Notably, 99% patients have received the inactivated COVID-19 vaccine (BBIBP-CorV or CoronaVac), of which 76% have received booster shots (third or even fourth doses). The interval between the last vaccination and the first BTI in our cohort was between six months and more than a year. The cohort consisted of 76 patients with mild symptoms, 31 with moderate symptoms. Detailed symptom comparisons and clinical information are presented in Tables 1 and S1. Moderate individuals showed more respiratory symptoms, such as high fever, fatigue, and cough, while digestive system problems were more prominent in mild cases than in moderate ones.

**Table 1:** Demographics and clinical characteristics of the research cohort

	Total (*n* = 107)	SARS-CoV-2 BTI Clinical classification: mild (*n* = 76)	SARS-CoV-2 BTI Clinical classification: moderate (*n* = 31)
Age, median (range), *y*	37 (8–66)	39 (20–66)	32 (8–56)
Sex, *n (%)*			
Male	42 (39)	32 (42)	10 (32)
Female	65 (61)	44 (58)	21 (68)
Symptoms, *n (%)*			
Fever	95 (89)	64 (84)	31 (100)
≥39°C	44 (46)	18 (24)	26 (84)
Fatigue	84 (79)	56 (74)	28 (90)
Cough	81 (76)	54 (71)	27 (87)
Pharyngalgia	60 (56)	37 (49)	23 (74)
Chest pain and tightness	33 (31)	18 (24)	15 (48)
Abdominalgia	10 (9)	8 (11)	2 (6)
Diarrhea	21 (20)	16 (21)	5 (16)
Nausea and vomiting	18 (17)	16 (21)	2 (6)
Feel sore and aching all over	64 (60)	46 (61)	18 (58)
Dizziness, headache	64 (60)	42 (55)	22 (71)
Taste loss	46 (43)	31 (41)	15 (48)
Hyposmia	32 (30)	22 (29)	10 (32)
Hearing loss	10 (9)	4 (5)	6 (19)
Tinnitus	9 (8)	3 (4)	6 (19)
Vaccination, *n (%)*	105 (98)	76 (100)	29 (94)
Strengthening needle	81 (77)	59 (78)	22 (76)

In addition, a small-scale COVID-19 epidemic occurred after 3–6 months since the first wave of Omicron BTI. Due to limited medical resources, most infected people were self-diagnosed using antigen test kits. In this cohort, 39 participants developed secondary BTI.

### Moderate group have higher and broader SARS-CoV-2-specific MBCs responses

We have previously reported strong neutralizing activity against Omicron BA.1, BA.2, BA.5, BF.7, and the previous prototype existed in vaccinated individuals with Omicron BA.5 and/or BF.7 BTI.^13^ However, it has lower neutralizing activity against Omicron sub lineages XBB, EG.5, JN.1, and KP.2. Compared with mild group, patients in moderate group had higher levels of nAbs. Based on the aforementioned findings, we further explored the SARS-CoV-2-specific MBCs levels in vaccinated individuals after Omicron BA.5 and/or BF.7 BTI. We used a flow cytometry-based B cell detection method to detect RBD-specific MBCs against prototype, Omicron BA.5, BF.7, and XBB. The specific gating strategy is shown in Figure S1. We found that there was no significant difference in total B cells between the mild and moderate groups, while the proportions of MBCs, activated MBCs, and plasma blasts were significantly increased in the moderate group at 1 month after BTI. Moreover, we found that prototype RBD^+^, BA.5 RBD^+^, and BF.7 RBD^+^ MBCs were significantly increased in patients with moderate group (*p* = 0.0029, *p* = 0.0007, *p* = 0.0328, respectively) at 1 month after BTI (Figures 1B and 1C). There was no significant difference in XBB RBD^+^ MBCs between the two groups. In addition, we compared the differences in specific MBCs in the total population in terms of gender, age, and number of symptoms at 1 month after BTI, and found that women, those under 35 years old and those with more than 7 symptoms, had higher specific memory B levels (Figure 1D).

**Figure 2:**
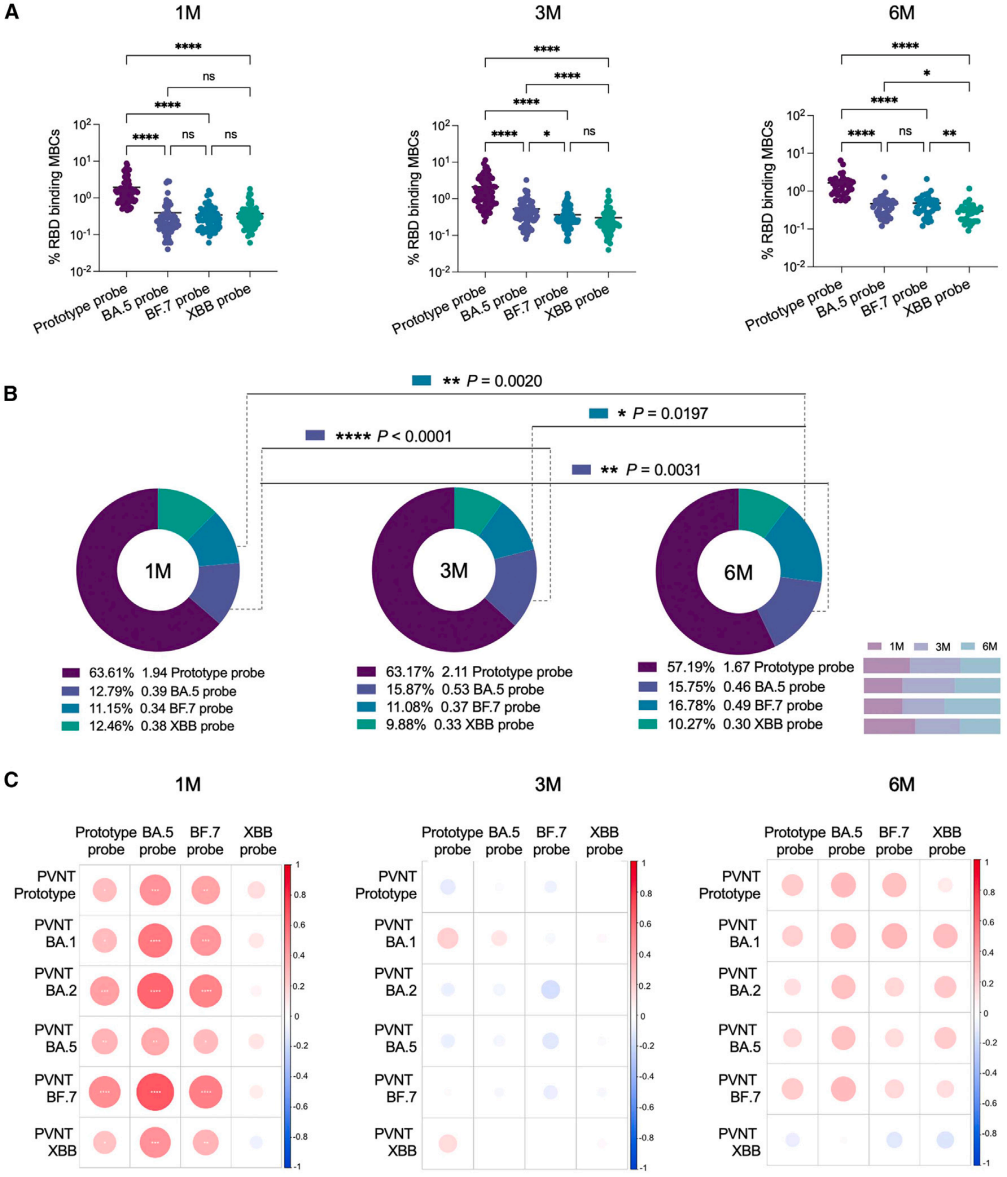
Progressive maturation of BA.5 RBD^+^ and BF.7 RBD^+^ MBCs (A) Scatterplot of comparison of four specific MBCs at 1M, 3M, and 6M after BTI. Data are represented as median. (B) Pie chart of comparison of four specific MBCs at 1M, 3M, and 6M after BTI, and comparison of the changes of specific MBCs over time. The percentages below the pie charts represent the proportion of RBD-specific MBCs. The numbers next to them represent the average. The rightmost number is the probe type. Bar chart shows the changes of specific MBCs with time points. (C) Pearson correlation matrices heatmap of the four probe-specific MBCs at 1M, 3M, and 6M after BTI and the nAbs results obtained at the corresponding time points for prototype, BA.1, BA.2, BA.5, BF.7, and XBB. Statistically significant correlations are indicated with an asterisk (∗). Two-tailed, nonparametric Dunn’s Kruskal-Wallis test was used for multiple comparisons. ^∗^*p* < 0.05; ^∗∗^*p* < 0.01; ^∗∗∗^*p* < 0.001; ^∗∗∗∗^*p* < 0.0001. ns, no significance. 1M, 1 month after Omicron BTI; 3M, 3 months after Omicron BTI; 6M, 6 months after Omicron BTI.

By comparing the four RBD-specific MBCs, it was found that prototype RBD^+^ MBCs at 1 month, 3 months, and 6 months (excluding the interference of secondary BTI) after BTI were significantly higher than those of the other three types (Figures 2A and 2B). By further comparing the levels of specific MBCs at 1 month, 3 months, and 6 months (excluding the interference of secondary BTI) after BTI, we found that although antibody levels decreased significantly with time,^13^ SARS-CoV-2-specific MBCs did not decrease. In particular, the levels of RBD-specific MBCs in Omicron-BA.5^+^ and BF.7^+^ were increased (Figure 2B). In addition, we conducted correlation analysis with four RBD-specific MBCs. There was a significant positive correlation between the RBD-specific MBCs of prototype^+^, Omicron-BA.5^+^, and BF.7^+^ and the nAbs at 1 month after BTI. No correlation between specific MBCs at 3 months and 6 months (excluding the interference of secondary BTI) after BTI and nAbs at the corresponding follow-up time points (Figure 2C).

### IgG^+^ and IgM^+^ MBCs after BTI

**Figure 3:**
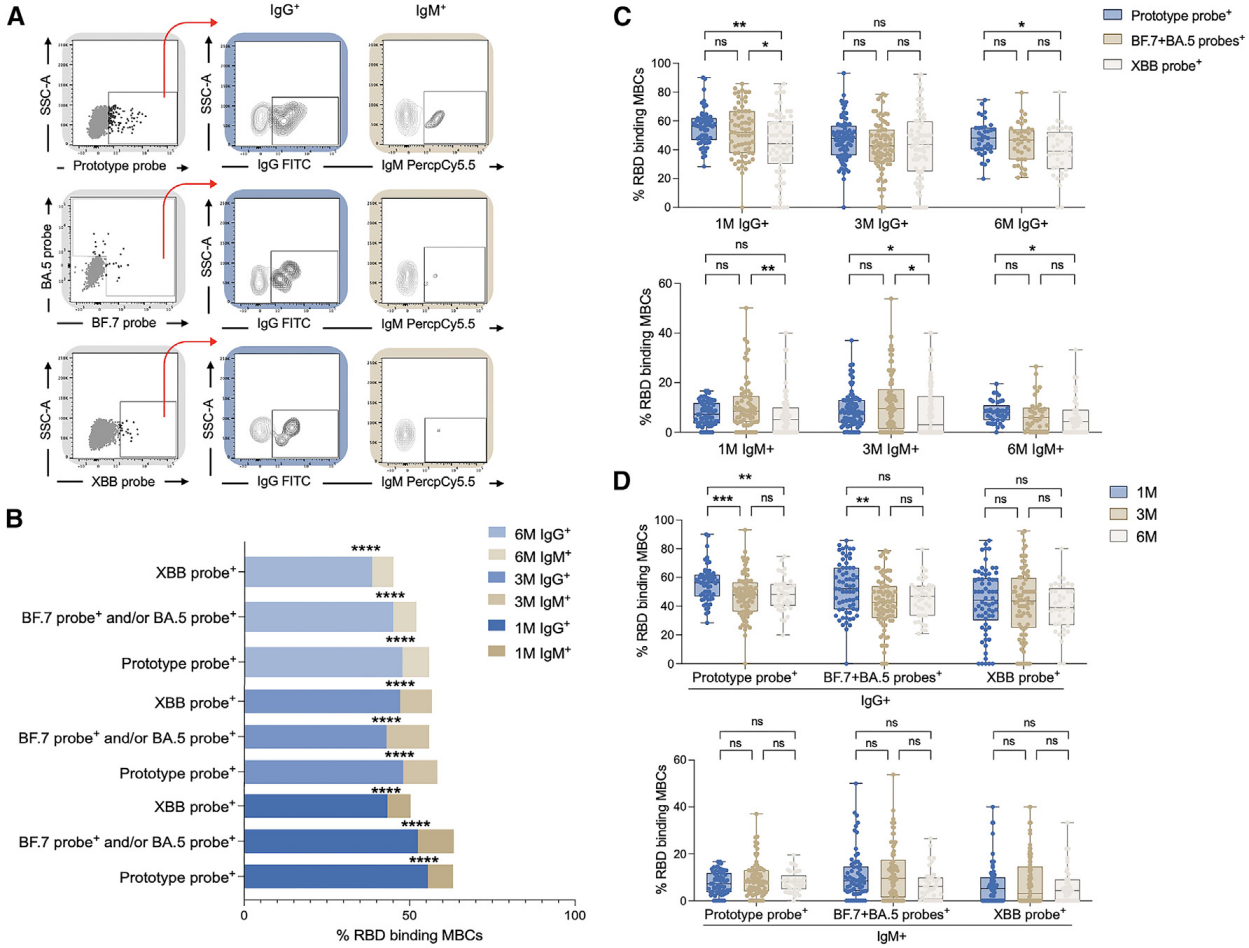
Homomorphic conversion of MBCs (A) Schematic diagram of IgG^+^ and IgM^+^ RBD-specific MBCs. (B) Comparison of various RBD-specific MBCs of IgG^+^ and IgM^+^ at 1M, 3M, and 6M after BTI. (C) Comparison of IgG^+^ and IgM^+^ in different RBD-specific MBCs. (D) Changes of IgG^+^ and IgM^+^ specific MBCs over time. Data are represented as median and interquartile range (IQR). Comparisons between two groups were performed using the two-tailed non-parametric Mann-Whitney test. Two-tailed, nonparametric Dunn’s Kruskal-Wallis test was used for multiple comparisons. ^∗^*p* < 0.05; ^∗∗^*p* < 0.01; ^∗∗∗^*p* < 0.001; ^∗∗∗∗^*p* < 0.0001. ns, no significance.

We further examined class-switched and non-class-switched MBCs after Omicron BA.5 and/or BF.7 BTI. It was found that IgG^+^ specific MBCs had significantly higher levels than IgM^+^, both early in the BTI and 6 months after the BTI (Figures 3A and 3B). Moreover, the BA.5 and/or BF.7 RBD^+^ specific MBCs of IgG^+^ and IgM^+^ and the prototype RBD^+^ specific MBCs of IgG^+^ and IgM^+^ were higher than the XBB RBD^+^ specific MBCs of IgG^+^ and IgM^+^ (Figure 3C). There was no difference in IgG and IgM levels between the mild and moderate groups (data not shown). In addition, we found that IgG^+^ but not IgM^+^ specific MBCs decreased with time (Figure 3D).

### Cross-reactive MBCs

**Figure 4:**
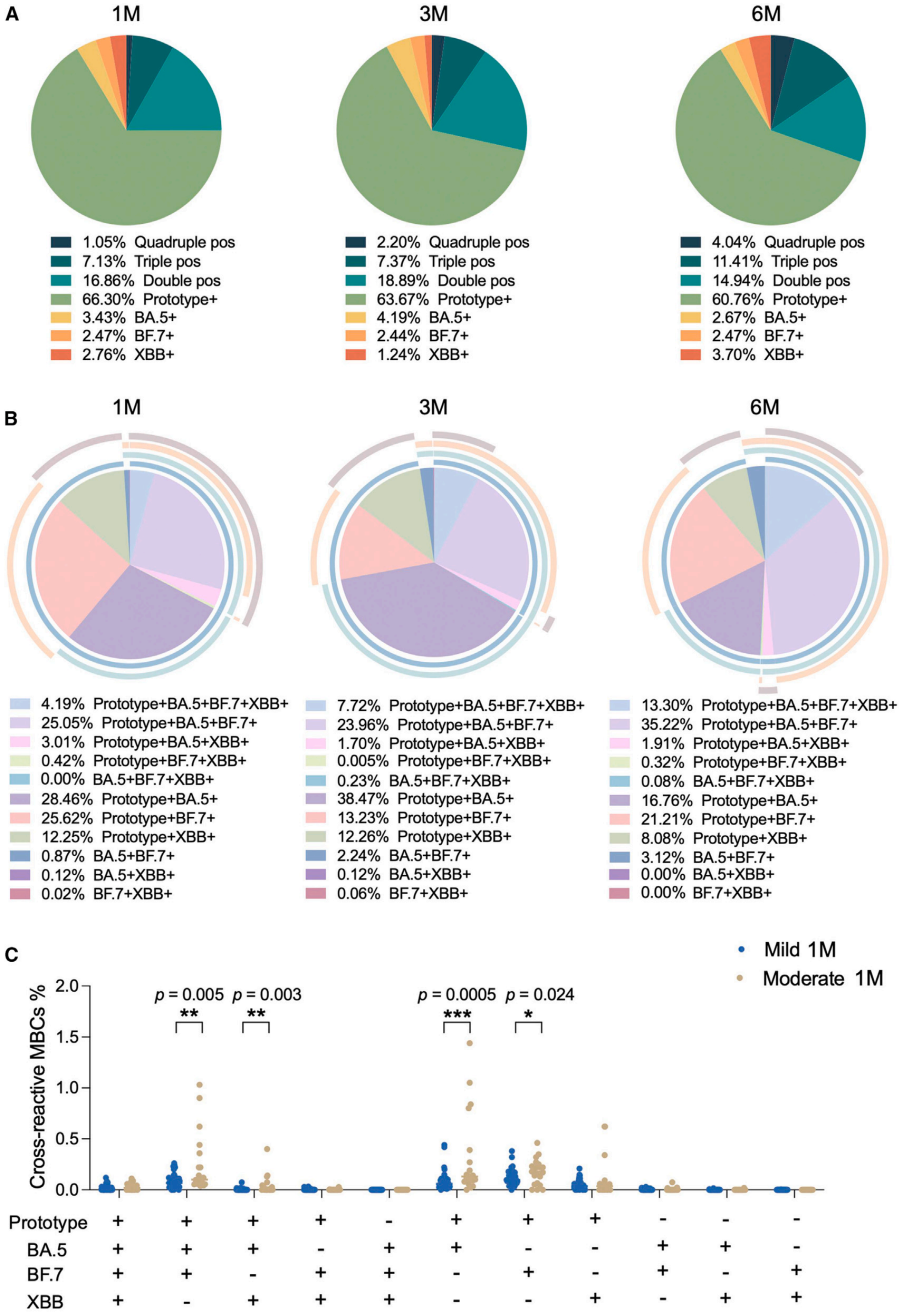
Cross-reactive MBCs (A) Quadruple-positive, triple-positive, double-positive, and single-positive MBCs at 1M, 3M, and 6M after BTI. (B) Various combinations of cross-reactive MBCs at 1M, 3M, and 6M after BTI. (C) Comparison of cross-reactive MBCs in mild and moderate groups at 1M after BTI. Data are represented as median. Comparisons between two groups were performed using the two-tailed non-parametric Mann-Whitney test. ^∗^*p* < 0.05; ^∗∗^*p* < 0.01; ^∗∗∗^*p* < 0.001.

Next, we analyzed the cross-reactive MBCs in our cohort. Using the fluorescence-activated cell sorting (FACS) gating strategy outlined in Figure S1A, four probe-positive gates were Boolean gated, resulting in various combinations of cross-reactive MBCs. Initially, we quantified and characterized the specific MBC subsets, including quadruple-positive, triple-positive, double-positive, and single-positive cells. Our analysis revealed that prototype-specific MBCs were dominant, further supporting the concept of immune imprinting (also known as “original antigenic sin”). Over the follow-up period, the frequency of single-positive prototype-MBCs decreased, while double-positive, triple-positive, and quadruple-positive MBCs demonstrated an increasing trend (Figure 4A). Subsequently, we focused on exploring the distribution of cross-reactive MBCs. The data clearly indicated that the most prevalent subsets were prototype^+^BA.5^+^BF.7^+^, prototype^+^BA.5^+^, and prototype^+^BF.7^+^ MBCs (Figure 4B). When comparing cross-reactive MBCs between mild and moderate groups at 1 month after BTI, the moderate group exhibited a stronger cross-reactive MBC response than the mild group. This difference was particularly notable in subsets such as prototype^+^BA.5^+^BF.7^+^, prototype^+^BA.5^+^XBB^+^, prototype^+^BA.5^+^, and prototype^+^BF.7^+^ (Figure 4C). These findings suggest that the first Omicron BA.5 and/or BF.7 BTI elicited a more robust immune response to the prototype antigen, likely due to pre-existing immunity induced by prior ancestral vaccination.

### SARS-CoV-2-specific MBCs may play a potential role in preventing secondary BTI

**Figure 5:**
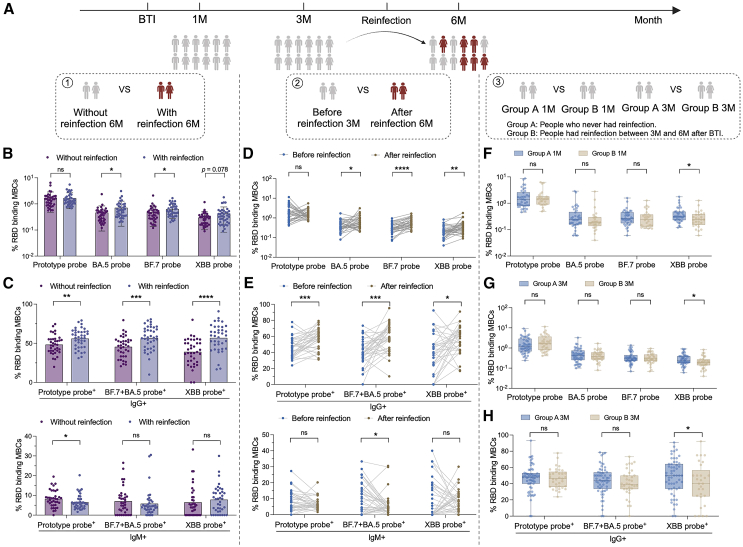
The role of SARS-CoV-2-specific MBCs in secondary BTI (A) Three analysis logics for secondary BTI. First, at the follow-up time point after secondary BTI, the two groups of people with and without secondary infection were compared. Second, the secondary infection group was compared before and after infection. Third, the immune differences between the people with and without secondary infection were traced back to the previous 1M and 3M. (B) Comparison of specific MBCs between people with and without secondary infection. Data are represented as the mean ± SD. (C) Comparison of IgG^+^ and IgM^+^ specific MBCs between people with and without secondary infection. Data are represented as the mean ± SD. (D) Paired comparison of specific MBCs before and after secondary infection. (E) Paired comparison of IgG^+^ and IgM^+^ specific MBCs before and after secondary infection. (F) Comparison of specific MBCs between people with secondary infection at 1M and those without secondary infection. Data are represented as median and interquartile range (IQR). (G) Comparison of specific MBCs between people with secondary infection at 3M and those without secondary infection. Data are represented as median and IQR. (H) Comparison of IgG^+^ and IgM^+^ specific MBCs between people with secondary infection at 3M and those without secondary infection. Data are represented as median and IQR. Comparisons between two groups were performed using the two-tailed non-parametric Mann-Whitney test and paired parametric t test. ^∗^*p* < 0.05; ^∗∗^*p* < 0.01; ^∗∗∗^*p* < 0.001; ^∗∗∗∗^*p* < 0.0001. ns, no significance.

During the follow-up period of this study, some subjects developed secondary BTI. In previous studies, we have revealed that the antibody level increased significantly after secondary infection, and the increase rate of XBB was much higher than that of other strains.^13^ We evaluated the possible impact of specific memory B on secondary infection through different aspects (Figure 5A). First, we compared the two groups of people with or without secondary BTI in samples taken 6 months after first BTI. Compared with subjects without secondary BTI, BA.5^+^ and BF.7^+^ MBCs were increased in the secondary BTI cohort. XBB^+^ MBCs was also increased in the trend (Figure 5B). Further comparison of the levels of IgG^+^ and IgM^+^ MBCs after reinfection showed that the levels of prototype^+^ IgG^+^ MBC and BF.7^+^ and/or BA.5^+^ IgG^+^ MBCs and XBB^+^ IgG^+^ MBCs increased, while the levels of prototype^+^ IgM^+^ MBCs decreased (Figure 5C). Secondly, we compared the specific MBCs of these people who had secondary BTI at 3 months and 6 months follow-up time. This was used to assess specific MBCs before and after secondary BTI. After reinfection, the levels of BA.5^+^, BF.7^+^, and XBB^+^ MBCs increased significantly (Figure 5D). Levels of IgG^+^ MBCs also increased, while levels of IgM^+^ MBCs decreased (Figure 5E). Importantly, we explored the function of specific memory B by comparing two groups of people with and without secondary BTI 1 month and 3 months after the first BTI. The results showed that the levels of XBB-specific MBCs at 1 month and 3 months in people who had secondary BTI were lower than those in people who did not have secondary BTI (Figures 5F and 5G). Levels of XBB^+^ IgG^+^ MBCs were also reduced in people who had secondary BTI (Figure 5H). Pre-existing Omicron-specific MBCs may play an important role in preventing secondary Omicron sub lineage infection.

### Immune characteristics of SARS-CoV-2 BTI with different levels of nAbs revealed by single-cell RNA sequencing

**Figure 6:**
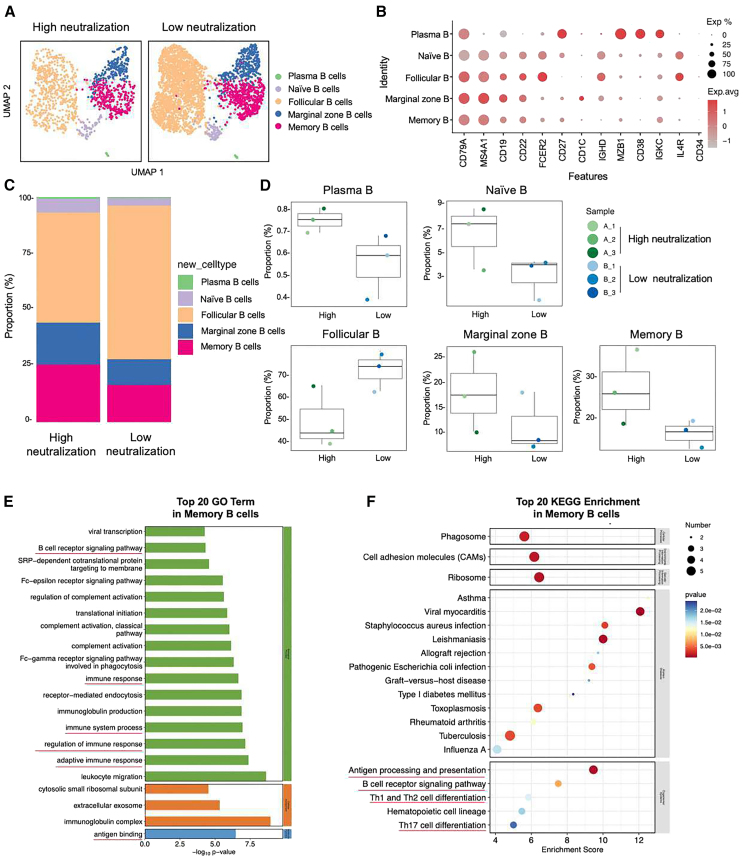
Immune characteristics of SARS-CoV-2 BTI with different levels of nAbs revealed by scRNA-seq (A) UMAP representation of B cells derived from High neutralization (*n* = 3) and Low neutralization (*n* = 3). Each dot corresponds to a single cell, colored according to cell type. (B) Dot plot of average expression and percentage of expressed cells of selected canonical markers in each labeled B cell subtype. (C) Bar plot showing B cell compositions at the single sample level. Average proportion of each cell type derived from two groups. (D) Comparison results of five B cell subsets in the two groups. The percentage of each cell cluster is calculated as: (the number of specific cell cluster) ⁄ (the number of B cells). (E) Gene enrichment analyses of the differentially expressed genes (DEGs) in the GO in memory B cells. GO terms are labeled with name and sorted by −log10 (*p* value). The top 20 enriched GO terms are shown. Green represents DEGs enriched in biological process-related signaling pathways. Orange represents cellular component. Blue represents molecular function. (F) DEGs enrichment analyses in the KEGG in MBCs. The horizontal axis is the enrichment score. Entries with larger bubbles contain more differential protein-coding genes. The color of the bubble changes from blue-white-yellow-red. The smaller the enrichment *p* value, the greater the significance.

We identified three samples with the highest nAbs levels and three samples with the lowest nAbs levels from a total of 66 samples collected one month after the first BTI. These samples were then subjected to single-cell transcriptome sequencing and BCR immunorepertoire analysis. The screening criteria involved ranking the sum of nAbs against prototype, BA.1, BA.2, BA.5, BF.7, and XBB in descending order to select the top three and bottom three samples. Furthermore, we separately ranked the sum of nAbs against prototype, BA.5, and BF.7, and the results were entirely consistent with the former ranking. Detailed information for the six selected samples is provided in Table S2. After Cell Ranger quality control, each sample yielded 6,619 to 10,531 high-quality cells. Following removal of doublets, multiplets, and apoptotic cells, the final cell count ranged from 5,848 to 9,031. Per cell, the average Unique Molecular Identifier (UMI) count was 5,501–7,142, genes were 2,116–2,551, and mitochondrial UMI ratio was 0.0230–0.0290. Clustering analysis grouped the cells into 16 clusters, identifying five main subpopulations: B cells, T cells, natural killer (NK) cells, platelets, and monocytes (Figures S2). This study conducted an in-depth analysis of B cells, further reduced dimensionality and clustered them, and identified the following five B cell subtypes based on the expression and distribution of classical B cell markers (Figures 6A and 6B). They are as follows: plasma B (*CD79A^+^* *MZB1^+^ CD38*^*+*^ *IGKC^+^*), naive B (*CD79A*^+^ *MS4A1*^+^ *CD19*^+^ *IL4R*^+^ *CD34*^*−*^), marginal zone B (*CD79A^+^ MS4A1^+^ CD19*^*+*^ *CD1C^+^*), follicular B (*CD79A^+^ MS4A1^+^ CD19*^*+*^ *CD22*^*+*^ *FCER2^+^ CD27*^*−*^), and memory B (*CD79A^+^ MS4A1^+^ CD27*^*+*^ *IGHD*^*−*^). We found that compared with the low-neutralization group in the BTI population, the high-neutralization group had a higher proportion of plasma B, naive B, marginal zone B, and memory B, while the proportion of follicular B decreased (Figures 6C and 6D). Based on the previous understanding of probe-specific MBCs, we will focus on some situations of MBCs in people with different neutralization levels. We first demonstrated the differentially expressed genes (DEGs) profiles of the five B cell subsets (Figure S3). To further understand the metabolic pathways, signaling pathways and biological processes that may be involved in the high and low neutralization groups on MBC subsets, we performed Kyoto Encyclopedia of Genes and Genomes (KEGG) and Gene Ontology (GO) enrichment analysis on DEGs. The results showed that DEGs were significantly enriched in GO biological processes such as BCR signaling pathway, immune response, immune system process and adaptive immune response, and GO molecular functions such as antigen binding (Figure 6E). In KEGG, signaling pathways such as Antigen processing and presentation, BCR signaling pathway, Th17 cell differentiation, Th1 and Th2 cell differentiation well explain the potential mechanism of B cell-induced humoral immunity on the production of nAbs (Figure 6F).

### Expanded B cells and specific rearrangements of V(D)J genes in high neutralization group

We further analyzed the BCR in combination with transcriptome data. We found that the low neutralization group had a higher proportion of B cells expressing BCR across all B cell clusters compared to the high neutralization group (Figure S4A). Clonal expansion was observed in follicular B, marginal zone B, and MBCs in both groups (Figure S4B). Although diversity indices (Chao1, Hill, and Shannon) were higher in the low neutralization group (Figure S4C), the level of BCR clonal amplification was significantly greater in the high neutralization group (Figure S4D). Given the pivotal role of CDR3 in antigen recognition, we focused on its characteristics. In contrast to T and B cells without immune expansion, which show a symmetric Gaussian distribution, the CDR3 amino acid sequence distribution in both groups was biased. We found that the amino acid distribution of CDR3β was similar between the two groups, though the ranges differed slightly: 6–33 amino acids in the high neutralization group and 6–35 amino acids in the low neutralization group. The most frequent CDR3 length in both groups was 11 amino acids, with a higher frequency in the high neutralization group (Figure S4E). Additionally, the top 10 CDR3 sequences differed between the groups (Figure S4F), with the high neutralization group showing large expansions in a few families, such as CQQYFSSPWTF, CNCRDSSTIVVF, and CAKSSAFDYW, unlike the more uniform expansion in the low neutralization group. VJ gene usage also differed slightly between the groups. B cells in the high neutralization group predominantly expressed IGHV3-64D, IGHV3-72, IGHV4-34, IGHV5-10-1, IGKV1-6, IGKV3-20, and IGKV2D-29 (Figure S4G). We also analyzed the top 10 V and J genes and all V-J gene pairs (Figures S5A and S5B), with the most common VJ pair in the high neutralization group being IGHV3-23 and IGHJ4, while in the low neutralization group, it was IGHV3-33 and IGHJ4 (Figure S5C).

## Discussion

The COVID-19 outbreak has stabilized, but the SARS-CoV-2 mutations continue to exist, and the characteristics of multiple variants co-epidemic are shown.^14^^,^^15^ At present, Omicron is still the most evolutionarily obvious SARS-CoV-2 variant of concern to date.^10^^,^^16^^,^^17^ We initially conducted a long-term follow-up of the population cohort after Omicron BTI and tested the nAbs levels of the population against multiple variants.^13^ The results showed that after Omicron BA.5 and/or BF.7 BTI, the population had strong neutralizing activity against Omicron BA.1, BA.2, BA.5, BF.7, and previous prototypes, and had no obvious neutralizing activity against the XBB, EG.5, JN.1, and KP.2 lineages after Omicron BA.5 and/or BF.7. Longitudinal follow-up study found that the antibody level decreased significantly over time, and secondary infection was inevitable in some people. Therefore, in-depth research on the changes in immune responses behind infection and designing vaccine strategies that can induce long-lasting and sufficiently broad immunity to protect against possible future viral variants are the current research focuses.

Long-lasting humoral immunity after infection depends on two distinct cell types that originate from germinal center (GC) reactions: long-lived plasma cells, which persistently produce antibodies, and MBCs, which possess the ability to proliferate and differentiate into antibody-secreting cells in response to a subsequent antigenic encounter.^18^^,^^19^^,^^20^ Our study found that after the first Omicron BA.5 and/or BF.7 BTI, the MBCs of prototype RBD^+^ was significantly higher than those of BA.5, BF.7, and XBB, suggesting induction of higher affinity for antigens that the host encountered previously in life. Sokal et al.^21^ revealed that BA.1 BTI mainly re-recruits existing cross-reactive MBCs, with limited new responses to BA.1 restricted epitope. Another study also suggested that the pool of long-lived MBCs imprinted with the vaccine was plastic enough to be remodeled by exposure to allogenic SARS-CoV-2 spikes. In bnt162b2 vaccinated individuals, the Omicron BA.1 BTI mainly expanded the extensive MBC bank targeting conserved S glycoproteins and RBD epitopes, rather than inducing strict Omicron BA.1 specific long-lived MBCs.^10^ Although antibody levels decreased significantly with time, SARS-CoV-2-specific MBC levels remains stable over time, and even increased in our follow-up study, the maturation of MBCs induced by antigen exposure was progressive. Similar phenomena have been reported in some previous studies.^22^^,^^23^^,^^24^ For example, Kaku et al.^23^ previously reported the maturation pattern of BA.1 specific MBCs after BA.1 BTI. Compared with the early stage, the proportion of BA.1 specific MBCs and cross-reactive MBCs between BA.1 and wild-type (WT) in the late stage samples increased significantly. Interestingly, there was no correlation between either antibodies or MBCs and XBB-specific MBCs one month after BTI. This further supports our epidemiological inference, that is, the main feature of our study cohort is that individuals who have completed inactivated vaccine vaccination have a BA.5 and/or BF.7 BTI. Our explanation is that in the initial epidemic background, XBB mutant has not appeared, and the production of neutralizing antibody is mainly based on the imprint left by vaccine immunization and the current antigen exposure. Antibodies are generated by B cells in response to viral infections or vaccinations. As crucial effector molecules capable of binding unprocessed antigens, they play a significant role in mitigating the effects of subsequent exposures.^25^ Furthermore, the involvement of MBCs in viral infections is multifaceted and dynamic, encompassing the production of cytokines, the presentation of antigens, and the secretion of antibodies.^26^^,^^27^^,^^28^^,^^29^ The role of the immune memory formed by the initial antigen exposure in the next encounter with the same antigen is clear, but little is known about the remodeling of mature MBCs by repeated viral mutation infection. During follow-up, a subset of participants experienced a second BTI, providing valuable insights into the functional role of MBCs. We formulated two analysis strategies for the longitudinal follow-up study. First, analyzing samples collected 6 months post-BTI-1 (1 month post-BTI-2) to quantify MBC dynamics post-reinfection. Results demonstrated that BTI-2 elicited significant expansion of Omicron-specific MBCs, predominantly targeting BA.5, BF.7, and XBB RBD epitopes. Second, using samples from 1 to 3 months after BTI-1 as the baseline for studying the role of pre-existing specific MBCs in responding to subsequent antigen exposure. We observed that participants without reinfection had higher baseline levels of XBB RBD^+^ MBCs at 1 and 3 months, suggesting that pre-existing specific MBC responses may help protect the body from subsequent antigen exposure. Yisimayi et al.^30^ mentioned that the activation and expansion of Omicron-specific naive B cells produced by Omicron for the first time can help to alleviate the immunoblotting induced by inactivated vaccine. Our results also show that repeated exposure to Omicron reduces the imprint left by the initial vaccine immunization and produces more MBCs specific to Omicron. In addition, MBCs produced after the first BTI play a potential protective role in the next exposure. This finding also further supports the inference of previous studies that the maturity of MBCs with high affinity and wide reactivity provides a basis for effective recall of SARS-CoV-2 variants in the future.^31^ Our study thus addresses the knowledge gap regarding MBC responses in repeated BTIs.

Based on our understanding of the levels of MBCs and nAbs after BTI, we further explore the potential mechanism of immune memory on nAbs production. Two groups with three high neutralization and three low neutralization samples were selected to study dominant cell types, gene profile, signaling pathway and clonal lineages. Our study revealed a higher proportion of MBCs in high neutralization group, and significant enrichment of DEGs in pathways related to antigen processing and immune regulation. We also revealed several differential genes that may affect antibody levels, such as IGHV3-48, IGKV3-20, IGKV1-9, HLA-DPA1, and so on. Interestingly, we have previously identified an IGHV3-53-encoded, RBD-targeting cross-neutralizing antibody, D6, with a VL derived from the IGKV1-9 lineage. D6 effectively neutralized multiple SARS-CoV-2 variants.^32^ Similarly, Dacon et al.^33^ reported that IGKV3-20-encoded antibodies can neutralize coronaviruses across different lineages. However, research on other variable genes remains limited, underscoring key directions for future studies. In addition, the diversity and specificity of B cell receptors are key to B cell function. When B cells are stimulated by an antigenic peptide, many B cells with the same BCR rapidly proliferate, a process known as BCR clonal amplification.^34^^,^^35^ We found that BCR amplification was more significant in the high neutralization group than in the low neutralization group, although the degree of diversity was higher in the low neutralization group. Higher BCR expression and diversity may indicate a more extensive but less specific immune response in low neutralization group. These B cells may remain in a more primitive or early activated state, with limited clonal expansion of specific antigens. The distribution of the VJ gene was slightly different between the two groups. Wang et al.^36^ suggested that several groups of VH genes, including VH3-30 and VH3-53, are overexpressed after Delta or Omicron BA.1 BTI. Our results comprehensively reveal the distribution of VJ genes on BCR after Omicron BA.5 and BF.7 BTI and compare the distribution differences between different neutralizing antibody levels. A distinct contrast was found that the high neutralizing antibody group shows a large expansion of a few families (CQQYFSSPWTF, CNCRDSSTIVVF, and CAKSSAFDYW), while the low neutralizing antibody group exhibits uniform, low expansion families. This finding suggests that focusing on a few specialized families may better support the development of urgently needed vaccines and monoclonal antibodies.

Collectively, our data show that repeated Omicron BTI effectively induced the generation and evolution of Omicron-specific MBCs. Our findings demonstrated repeated Omicron BTI can subvert the impact of prototype original antigenic sin, leading to a more diverse and potent variant specific B cell response. The data generated in our work may be useful for the development of the next generation of vaccines and therapeutics.

### Limitations of the study

Nevertheless, our study has some limitations. We did have serum samples from 1, 3, and 6-month time points after BTI, but we didn’t collect nasopharyngeal swabs from subjects during their infection period for sequence testing. In addition, we lack baseline data and basic immune characteristics before first BTI. The sample size of single cell detection is limited, and expanding the sample size may be more conducive to revealing the role of MBCs. Future research will aim to address these limitations.

## Resource availability

### Lead contact

Further information and requests for resources and reagents should be directed to and will be fulfilled by the lead contact, Yiming Shao (yishao16@zju.edu.cn).

### Materials availability

This study did not generate new materials.

### Data and code availability

• All data supporting the findings in this study are available within the article or can be obtained from the lead contact upon request.
• This paper does not report original code.
• Any additional information required to reanalyze the data reported in this paper is available from the lead contact upon request.
• scRNAseq data have been deposited at CNSA public data repository and are publicly available as of the date of publication. Accession number (CNP0006986) is listed in the key resources table.

## Acknowledgments

The authors thank OE Biotech Co., Ltd. (Shanghai, China) for their assistance on single-cell RNA sequencing and Wenxing Li and Nan Hua for technical support and bioinformatics analysis separately in this study. This study was supported by grants from the 10.13039/501100001809National Natural Science Foundation of China (U20A20362); The First Affiliated Hospital, 10.13039/501100004835Zhejiang University School of Medicine, 10.13039/501100015360State Key Laboratory of Infectious Disease Prevention and Control Grant (no. 2021SKLID304 and no. 2020SKLID102); and Science and Technology Project of Beijing under Grant (Z211100002521024).

## Author contributions

Conceptualization, Q.C. and Y.S.; methodology, K.L.; validation, Q.C., Q.H., and L.R.; formal analysis, Q.C., and K.L.; investigation, J.W., S.W., and X.L.; writing – original draft, Q.C.; writing – review & editing, Q.C., Y.L., J.H., and D.L.; visualization, Y.S.; supervision, D.L., and Y.S.; funding acquisition, Y.S.

## Declaration of interests

The authors declare no competing interests.

## STAR★Methods

### Key resources table

**Table:** 

REAGENT or RESOURCE	SOURCE	IDENTIFIER
**Antibodies**		

CD3-PB	BD	Cat# 558117; RRID: AB_397038
CD8-PB	BD	Cat# 558207; RRID: AB_397058
CD14-PB (eflur450)	eBioscience	Cat# 48-0149-42; RRID: AB_1272050
CD19-BV510	BD	Cat# 302242; RRID: AB_2561668
CD20-ECD	Beckman	Cat# IM3607U; RRID: AB_10645191
IgG-FITC	BD	Cat# 555786; RRID: AB_396121
IgM-PercpCy5.5	BD	Cat# 561285; RRID: AB_10611998
CD27-APC-Cy7	BD	Cat# 560222; RRID: AB_1645474
IgD-PE-Cy7	Biolegend	Cat# 348210; RRID: AB_10680462
CD38-BV650	BD	Cat# 740574; RRID: AB_2740275
Streptavidin-PE-Cy5	Biolegend	405205
Streptavidin-APC	Biolegend	405207
Streptavidin-PE	Invitrogen	S21388
Streptavidin-BV605	Biolegend	405229

**Chemicals, peptides, and recombinant proteins**		

RPMI 1640	Corning	10-040-Cva
Bezonase	Sigma	E1014-25KU
Fetal bovine serum (FBS)	ExCell bio	FND500
Penicillin/streptomycin	Gibco	15140-122
Phosphate buffered solution (PBS)	Corning	21-040-CVC
Ficoll	Cytiva	10340750
Biotinylated SARS-CoV-2 Spike RBD Protein, His, Avitag™ (BA.4&BA.5/Omicron) (MALS verified)	ACRO Biosystems	SPD-C82Ew
Biotinylated SARS-CoV-2 Spike RBD Protein, His, Avitag™ (BF.7&BA.4.6/Omicron) (MALS verified)	ACRO Biosystems	SPD-C82E1
Biotinylated SARS-CoV-2 Spike RBD Protein, His, Avitag™ (XBB/Omicron) (MALS verified)	ACRO Biosystems	SPD-C82Q1
Biotinylated SARS-CoV-2 Spike RBD Protein, His, Avitag™ (BA.4&BA.5/Omicron) (MALS verified)	ACRO Biosystems	SPD-C82Ew

**Critical commercial assays**		

LIVE/DEAD® Fixable Blue Dead Cell Stain Kit	Invitrogen	L23105

**Deposited data**		

scRNAseq dataCN	CNSA public data repository	CNP0006986
KEGG	Kyoto Encyclopedia of Genes and Genomes	https://www.genome.jp/kegg/pathway.html
GO	Gene Ontology	https://geneontology.org/

**Software and algorithms**		

GraphPad Prism	Version 9	Graphpad.com
Flowjo	Version 10.8.1	Flowjo.com
SPSS	Version 26	SPSS.com
R	V4.2.2	https://www.npackd.org/p/r/4.2.2/

### Experimental model and study participant details

107 participants with Omicron BTI and had no history of COVID-19 were recruited, including 31 experienced moderate symptoms, while 76 had mild symptoms, classified according to the 10th Edition of China's National Health Commission's guidelines. Serum samples were collected from January to July 2023 at three intervals: 1, 3, and 6 months post-BTI. Plasma and peripheral blood mononuclear cells (PBMCs) were separated from blood by Ficoll density gradient centrifugation. This study was conducted in compliance with the Declaration of Helsinki and received approval from the Ethics Committee of Zhejiang University School of Medicine (Ref. Nos. 20230110 and 20230787). All participants provided informed consent.

### Method details

#### Construction of RBD probe

Antigen-specific MBCs were detected using recombinant biotinylated antigens tetramerized with fluorophore-conjugated streptavidin (SA). For detection of MBCs that recognize prototype, BA.5, BF.7 and XBB RBD, biotinylated antigens to SA were mixed in the following combinations: Biotinylated SARS-CoV-2 Spike RBD Protein, His, Avitag™ (prototype) (ACRO Biosystems, Cat #SPD-C82E9) with SA-PE-Cy5 (Biolegend), Biotinylated SARS-CoV-2 Spike RBD Protein, His, Avitag™ (BA.5) (ACRO Biosystems, Cat #SPD-C82Ew) with SA-PE (Invitrogen), Biotinylated SARS-CoV-2 Spike RBD Protein, His, Avitag™ (BF.7) (ACRO Biosystems, Cat #SPD-C82E1) with SA-APC (Biolegend), and Biotinylated SARS-CoV-2 Spike RBD Protein, His, Avitag™ (XBB) (ACRO Biosystems, Cat #SPD-C82Q1) with SA-BV605 (Biolegend). 1/5 of the molar equivalent of the SA-fluorophores was added to biotinylated RBD and incubated on ice for 20 min with gentle rocking. The above steps were repeated 4 times, and finally the labeled probes were stored at 4°C, avoiding light.

#### Evaluation of SARS-CoV-2 RBD-specific MBCs responses by flow cytometry

PBMCs was thawed and resuspended in a pre-warmed RPMI-1640 containing 1% PS, 10% FBS and 50 IU/mL benzonase. After washing with FACS buffer (2% BSA/1mM EDTA in 1X PBS), the PBMCs were stained with a LIVE/DEAD dye (Invitrogen) on ice for 20 min. After washing, the PBMCs were stained with an antibody cocktail containing anti-CD14-Pacific Blue (eBioscience), anti-CD20-ECD (Beckman), anti-IgD-PE-Cy-7 (Biolegend), anti-CD3-Pacific Blue, anti-CD8-Pacific Blue, anti-CD19-BV510, anti-IgG-FITC, anti-IgM-PercpCy5.5, anti-CD27-APC-Cy7, anti-CD38-BV650 (all from BD Biosciences), and RBD-Probes. Then incubated for 1h on ice. After washing two times with FACS buffer, samples were analyzed using a Fortessa LSR flow cytometer (LSRFortessa™, BD) and performed data analysis using FlowJo (version 10.8.1).

#### Single-cell 5′ mRNA and VDJ library construction and sequencing

First, cells were counted using Countess®II to accurately adjust the cell concentration to 1×10^6^/mL. Cells were mixed with enzymes and gel beads containing UMI sequences and encapsulated in droplets through a microfluidic chip, where cell lysis occurred, RNA was captured and reverse transcribed to generate cDNA. Using the 5' library construction method, mRNA was reverse transcribed using a poly(T) primer to attach the sequence to the 5' end of the cDNA, thereby enriching the V(D)J region. Random fragmentation and PCR amplification ultimately formed a DNA library, ensuring high-quality data for downstream analysis.

#### Single-cell RNA-seq data processing

The FASTQ files were aligned to the GRCh38 human genome using Cell Ranger (version 8.0.1) from 10x Genomics, with UMI counts summarized per barcode. Quality filtering removed low-quality cells and potential multiplets based on criteria: gene count (< 200), UMI count (< 1000), log10GenesPerUMI (< 0.7), and mitochondrial RNA content (> 10%). Gene expression data were normalized with the "LogNormalize" method in Seurat (version 4.0.0), followed by HVG identification (top 2000), dimensionality reduction (PCA), clustering (graph-based method), and visualization (UMAP). Cluster markers were identified using the FindAllMarkers function (test. use = presto), and DEGs were filtered with a threshold of *P* < 0.05 and fold change > 1.2. GO and KEGG enrichment analyses were conducted on DEGs using R (version 4.0.3).

#### Single-cell 5′ V(D)J data processing

A CSV file specified paths for the 5' gene expression, feature barcode, and V(D)J libraries. Data were processed with alignment, filtering, barcode, and UMI counting, with V(D)J libraries undergoing sequence assembly and clonotype pairing. Gene expression-based cell calling ensured only relevant cells were included in the V(D)J analysis, enhancing accuracy. The "cellranger multi" command generated single-cell feature counts, V(D)J sequences, and annotations, using reference genomes refdata-gex-GRCh38-2024-A and refdata-cellranger-vdj-GRCh38-alts-ensembl-7.0.0. Clonotype information was integrated into Seurat-based single-cell analyses, with VDJtools (version 1.1.4) supporting downstream diversity analysis.^37^^,^^38^

### Quantification and statistical analysis

Data and statistical analyses were performed in Prism (version 9.5.0) and SPSS (26). Two-tailed nonparametric Mann–Whitney U test and Kruskal–Walli’s test were performed on numerical data. The statistical tools and methods for each analysis are explicitly described with the results or detailed in the figure legends. It is important to note that all boxplots in the manuscript are presented using the median and interquartile range (IQR). The line in the center of all scatter plots represents the median. All bar scatter plots are presented using the mean with standard deviation (SD). *P* < 0.05 were considered statistically significant.

## Supplemental information

Document S1. Figures S1–S5 and Table S2

Table S1. Detailed clinical information of the research cohort
