# Supplementary material for: Efficient boosting of Omicron-reactive memory B cells after breakthrough infection protects from repeated exposure
Source: iScience. 2025 Mar 25;28(4):112278. doi: 10.1016/j.isci.2025.112278 (PMC12013488; doi:10.1016/j.isci.2025.112278)
Supplement: Document S1. Figures S1–S5 and Table S2 [file mmc1.pdf]

iScience, Volume *28*

Supplemental information

Efficient boosting of Omicron-reactive memory B cells after breakthrough infection protects from repeated exposure

Qingfei Chu, Kang Li, Qianxin He, Li Ren, Jiguo Wang, Shuo Wang, Xiaojing Liu, Ying Liu, Jiangshan He, Dan Li, and Yiming Shao

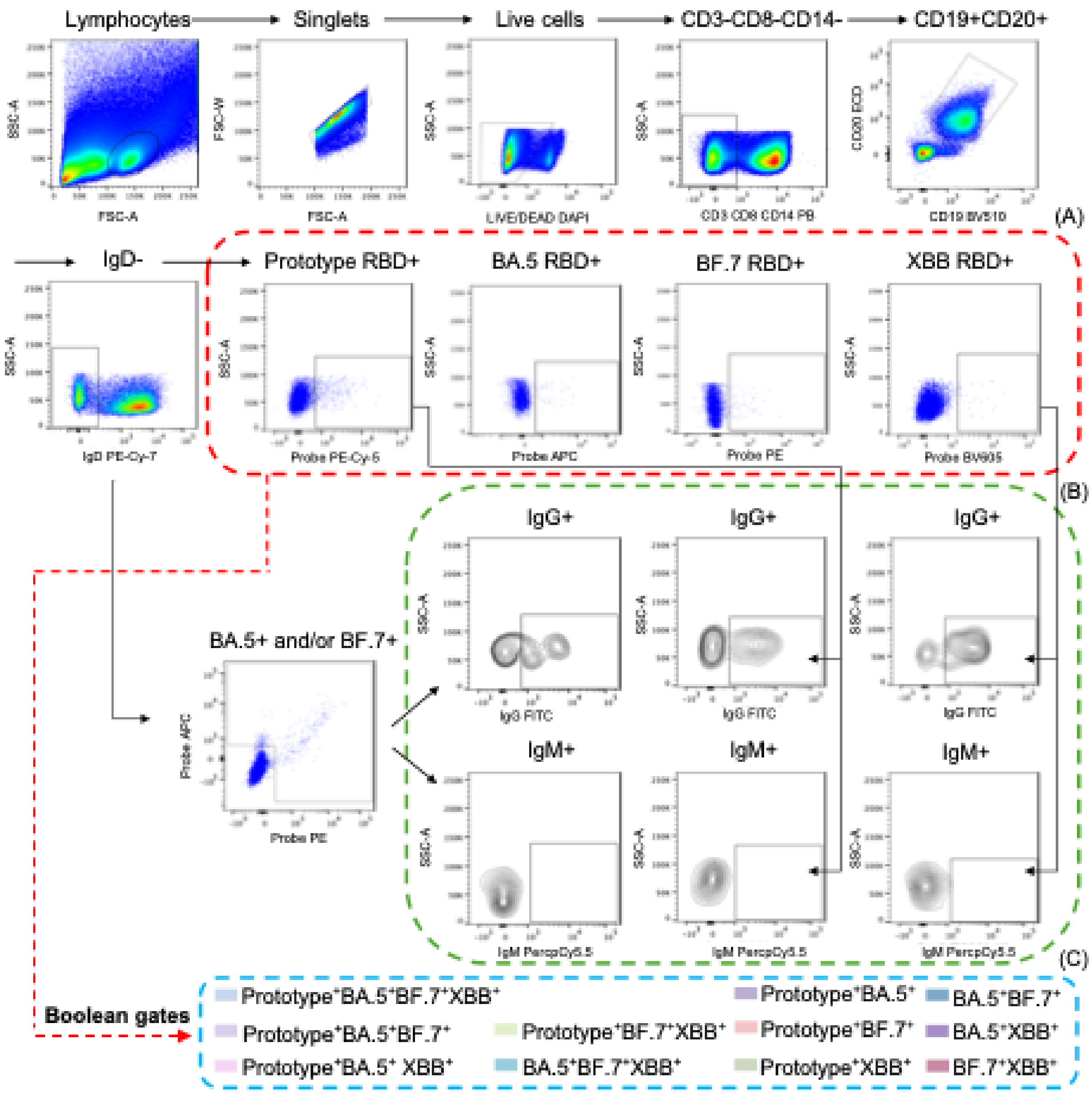

Lymphocytes
Singlets
Live cells
CD3-CD8-CD14-
CD19+CD20+
SSC-A
FSC-A
FSC-W
LIVE/DEAD DAPI
CD3 CD8 CD14 PB
CD20 ECD
CD19 BV510
(A)
IgD-
Prototype RBD+
BA.5 RBD+
BF.7 RBD+
XBB RBD+
IgD PE-Cy-7
Probe PE-Cy-5
Probe APC
Probe PE
Probe BV605
(B)
IgG+
IgG+
IgG+
BA.5+ and/or BF.7+
Probe APC
Probe PE
IgG FITC
IgM+
IgM+
IgM+
IgM PercpCy5.5
(C)
Boolean gates
Prototype+BA.5+BF.7+XBB+
Prototype+BA.5+
BA.5+BF.7+
Prototype+BA.5+BF.7+
Prototype+BF.7+XBB+
Prototype+BF.7+
BA.5+XBB+
Prototype+BA.5+ XBB+
BA.5+BF.7+XBB+
Prototype+XBB+
BF.7+XBB+

Figure S1. FACS gating strategy for SARS-CoV-2 RBD-specific B cell staining

(A) Representative FACS gating strategy to determine frequencies of Prototype^{+}, BA.5^{+}, BF.7^{+} and XBB^{+} MBCs.

(B) Representative FACS gating strategy to determine frequencies of IgG^{+} and IgM^{+} MBCs.

(C) Using the FACS gating strategy outlined in (A), four RBD-positive gates were Boolean gated, resulting in various combinations of cross-reactive MBCs.

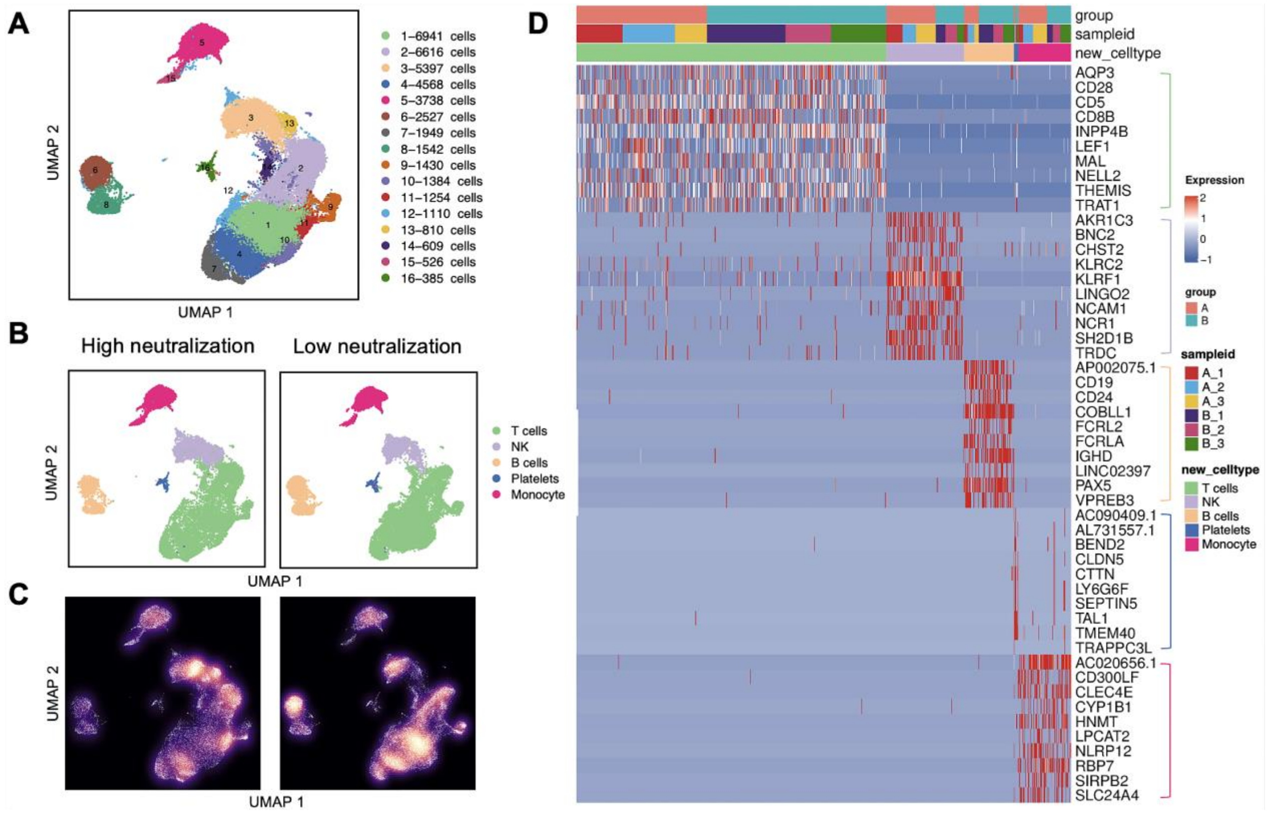
A
UMAP 2
UMAP 1
1–6941 cells
2–6616 cells
3–5397 cells
4–4568 cells
5–3738 cells
6–2527 cells
7–1949 cells
8–1542 cells
9–1430 cells
10–1384 cells
11–1254 cells
12–1110 cells
13–810 cells
14–609 cells
15–526 cells
16–385 cells
B
High neutralization
Low neutralization
UMAP 2
UMAP 1
T cells
NK
B cells
Platelets
Monocyte
C
UMAP 2
UMAP 1
D
group
sampleid
new_celltype
AQP3
CD28
CD5
CD8B
INPP4B
LEF1
MAL
NELL2
THEMIS
TRAT1
AKR1C3
BNC2
CHST2
KLRC2
KLRF1
LINGO2
NCAM1
NCR1
SH2D1B
TRDC
AP002075.1
CD19
CD24
COBLL1
FCRL2
FCRLA
IGHD
LINC02397
PAX5
VPREB3
AC090409.1
AL731557.1
BEND2
CLDN5
CTTN
LY6G6F
SEPTIN5
TAL1
TMEM40
TRAPPC3L
AC020656.1
CD300LF
CLEC4E
CYP1B1
HNMT
LPCAT2
NLRP12
RBP7
SIRPB2
SLC24A4
Expression
2
1
0
−1
group
A
B
sampleid
A_1
A_2
A_3
B_1
B_2
B_3
new_celltype
T cells
NK
B cells
Platelets
Monocyte

Figure S2. Single cell sequencing results

(A) Dimensionality reduction clustering result graph.

(B) Cell type identification results in high and low neutralization groups.

(C) High-density maps of cell type identification in high and low neutralization groups.

(D) Heat maps of Top10 Marker gene expression in each cell subpopulation.

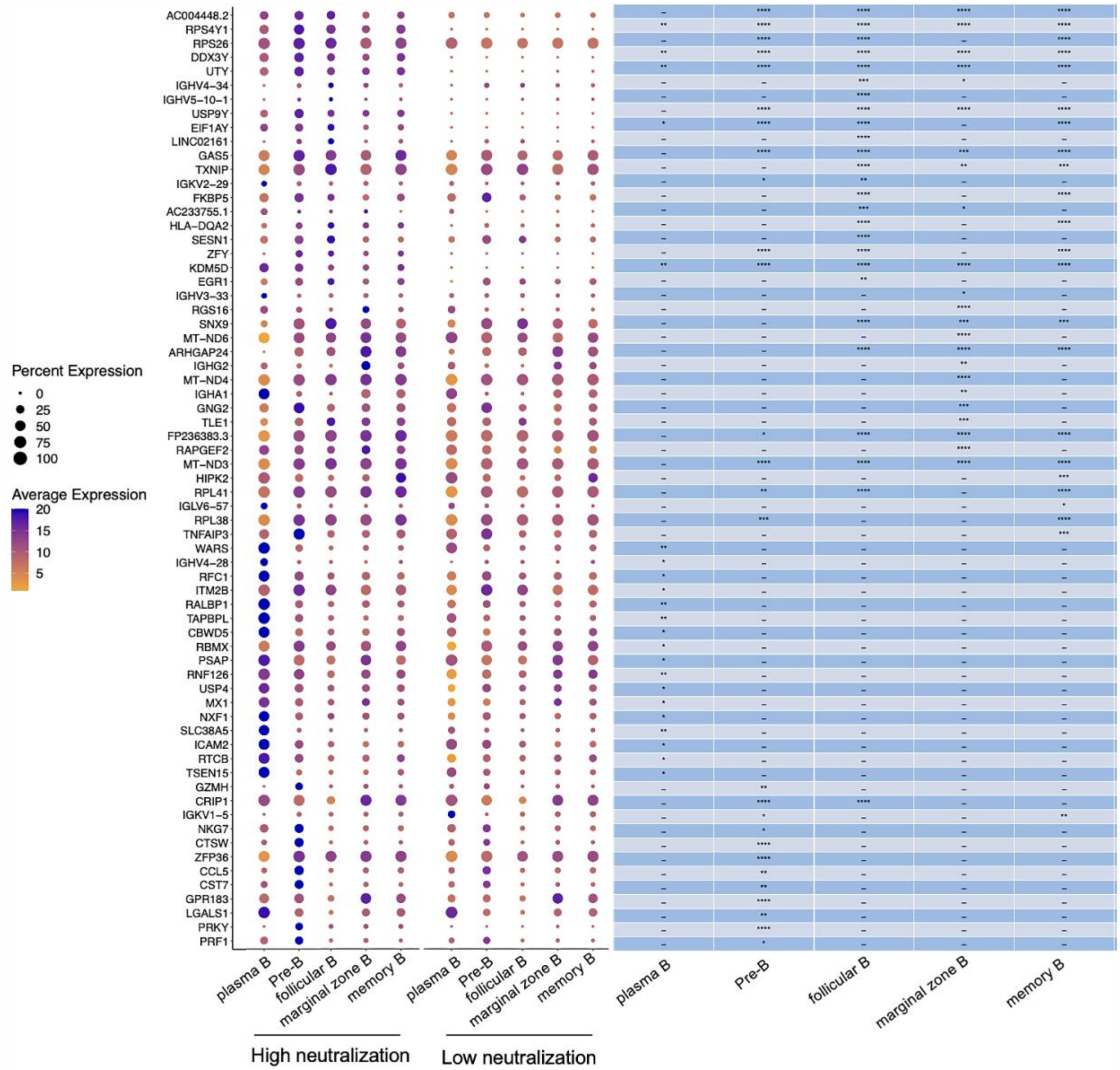
AC004448.2
RPS4Y1
RPS26
DDX3Y
UTY
IGHV4−34
IGHV5−10−1
USP9Y
EIF1AY
LINC02161
GAS5
TXNIP
IGKV2−29
FKBP5
AC233755.1
HLA−DQA2
SESN1
ZFY
KDM5D
EGR1
IGHV3−33
RGS16
SNX9
MT−ND6
ARHGAP24
IGHG2
MT−ND4
IGHA1
GNG2
TLE1
FP236383.3
RAPGEF2
MT−ND3
HIPK2
RPL41
IGLV6−57
RPL38
TNFAIP3
WARS
IGHV4−28
RFC1
ITM2B
RALBP1
TAPBPL
CBWD5
RBMX
PSAP
RNF126
USP4
MX1
NXF1
SLC38A5
ICAM2
RTCB
TSEN15
GZMH
CRIP1
IGKV1−5
NKG7
CTSW
ZFP36
CCL5
CST7
GPR183
LGALS1
PRKY
PRF1
Percent Expression
0
25
50
75
100
Average Expression
20
15
10
5
plasma B
Pre-B
follicular B
marginal zone B
memory B
High neutralization
Low neutralization

Figure S3. Differential gene profiles of five B cell subsets

The top 20 DEGs of each cell cluster were determined according to the average log FC value, and the top 20 DEGs of all cell clusters in the two groups were summarized to obtain a total of 67 DEGs. The size of the dots indicates the percentage of cell expression, and the color indicates the level of expression on a logarithmic scale. The right side of the figure shows the significance of differential expression between the two groups. The unmatched bilateral t test was used for statistical analysis. **P* < 0.05; ***P* < 0.01; ****P* < 0.001; *****P* < 0.0001. -, no significance or not detected.

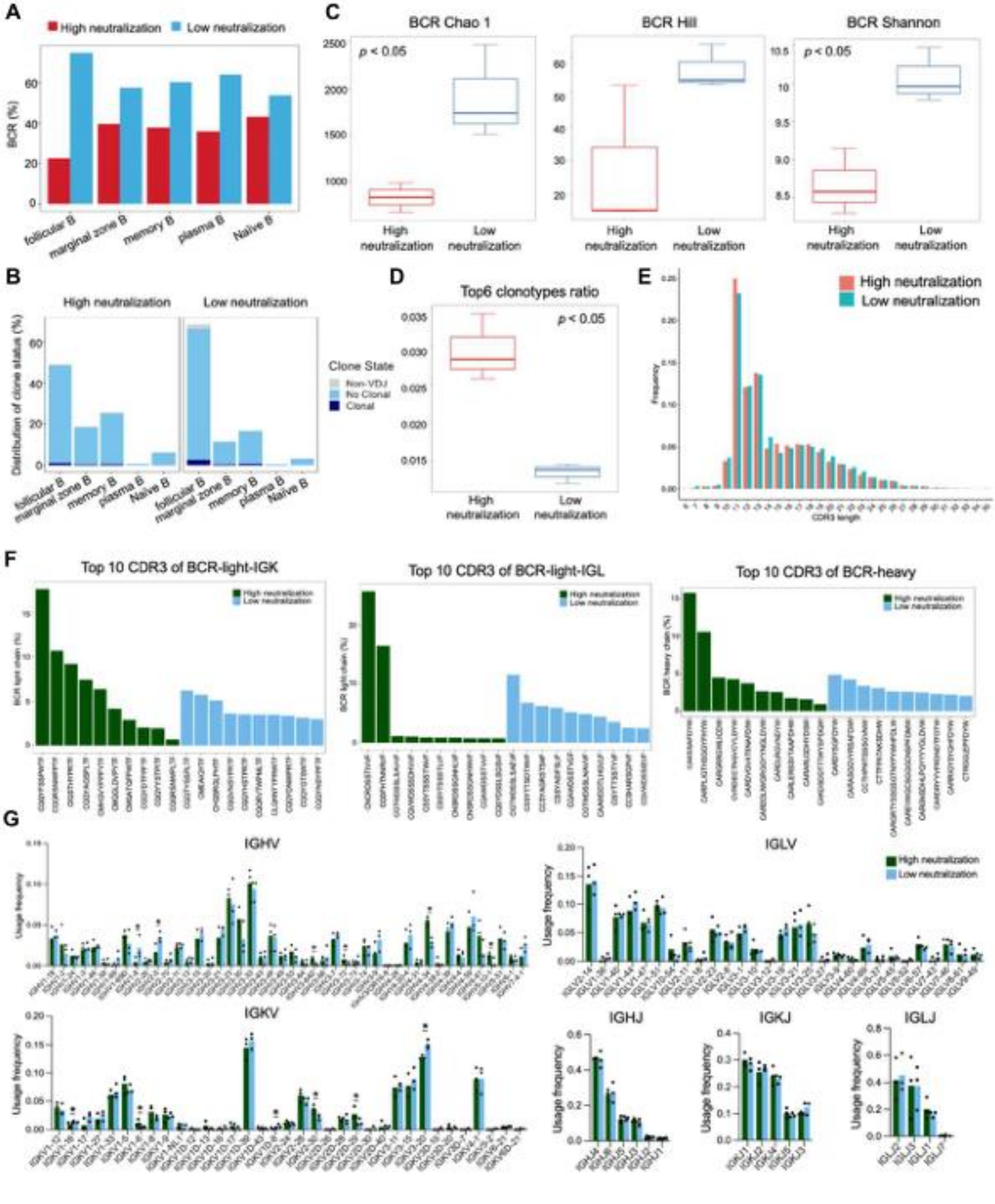
A
High neutralization
Low neutralization
BCR (%)
follicular B
marginal zone B
memory B
plasma B
Naive B
B
High neutralization
Low neutralization
Distribution of clone status (%)
Clone State
Non-VDJ
No Clonal
Clonal
C
BCR Chao 1
BCR Hill
BCR Shannon
p < 0.05
D
Top6 clonotypes ratio
E
Frequency
CDR3 length
F
Top 10 CDR3 of BCR-light-IGK
Top 10 CDR3 of BCR-light-IGL
Top 10 CDR3 of BCR-heavy
G
IGHV
IGLV
IGKV
IGHJ
IGKJ
IGLJ
Usage frequency

Figure S4. Expanded B cells and specific rearrangements of V(D)J genes in high neutralization group

(A) Bar plot showing percentage of B cells expressing BCR in each B cell cluster in High neutralization and Low neutralization groups.

(B) Bar plot showing the distribution of the clone state of B cells in each cluster of two groups. Clonotype copy number greater than 2 is clonal while clonotype copy number equal to 1 is nonclonal, and no clonotype represent non-BCR is marked as non-VDJ.

(C) BCR Chao, Hill, and Shannon diversity between two groups.

(D) BCR clonal expansion between two groups.

(E) The comparison of CDR3β length distribution of clonally expanded BCRs in two groups.

(F) The top ten CDR3 usages of protein sequences of heavy chain and light chain are shown between two groups.

(G) Graph shows relative abundance of human IGH-V, IGL-V, IGK-V, IGH-J, IGK-J and IGL-J genes between two groups.

Comparisons between two groups were performed using the two-tailed non-parametric Mann-Whitney test. *P < 0.05.

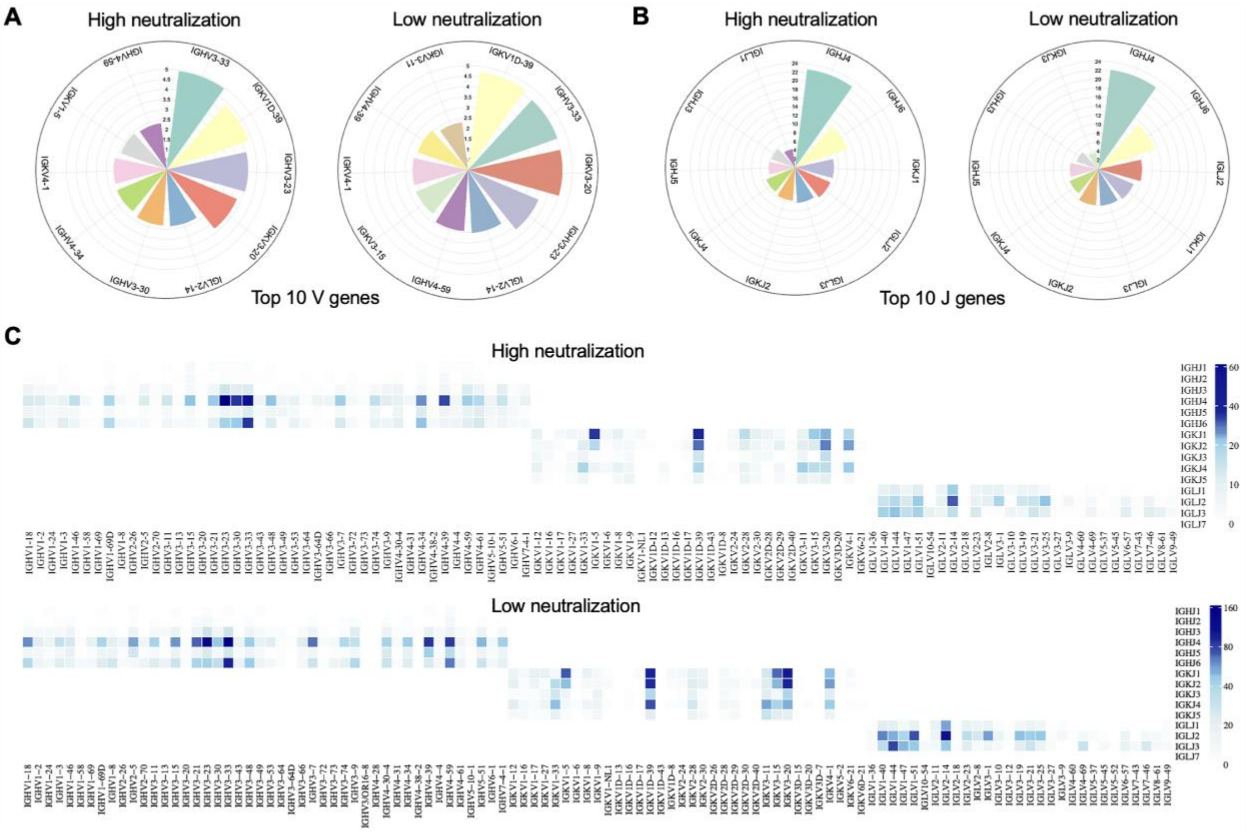
A
High neutralization
Low neutralization
IGHV3-33
IGKV1D-39
IGHV3-23
IGKV3-20
IGLV2-14
IGHV3-30
IGHV4-34
IGKV4-1
IGKV1-5
IGHV4-59
IGKV1D-39
IGHV3-33
IGKV3-20
IGHV3-23
IGLV2-14
IGHV4-59
IGKV3-15
IGKV4-1
IGHV4-39
IGKV3-11
Top 10 V genes
B
High neutralization
Low neutralization
IGHJ4
IGHJ6
IGKJ1
IGLJ2
IGLJ3
IGKJ2
IGKJ4
IGHJ5
IGHJ3
IGLJ1
IGHJ4
IGHJ6
IGLJ2
IGLJ1
IGLJ3
IGKJ2
IGKJ4
IGHJ5
IGHJ3
IGKJ3
Top 10 J genes
C
High neutralization
Low neutralization
IGHJ1
IGHJ2
IGHJ3
IGHJ4
IGHJ5
IGHJ6
IGKJ1
IGKJ2
IGKJ3
IGKJ4
IGKJ5
IGLJ1
IGLJ2
IGLJ3
IGLJ7

Figure S5. VJ gene use and gene pairing in two groups

(A) Top 10 V genes. The same color indicates the same V gene.

(B) Top 10 J genes. The same color indicates the same J gene.

(C) Heat maps showing IGH/K/L VJ genes rearrangement in two groups.

Table S2. Detailed information of six selected samples for scRNA seq

Group	Sample number	Sex	Age	$pVNT_{50}$ to Prototype	$pVNT_{50}$ to BA.1	$pVNT_{50}$ to BA.2	$pVNT_{50}$ to BA.5	$pVNT_{50}$ to BF.7	$pVNT_{50}$ to XBB
A High neutralization	A-1	Female	24	4619	2945	9000	9000	7290	407
	A-2	Female	29	5906	3632	9000	3547	9000	600
	A-3	Female	55	4812	3346	5758	5695	5108	343
B Low neutralization	B-1	Female	47	139	94	147	78	138	10
	B-2	Male	24	166	69	139	82	120	36
	B-3	Female	46	82	41	269	224	296	10
Asymp. Sig. (2-tailed) (A vs B)				$P < 0.05$	$P < 0.05$	$P < 0.05$	$P < 0.05$	$P < 0.05$	$P < 0.05$
